# Identity Diffusion and Extremist Attitudes in Adolescence

**DOI:** 10.3389/fpsyg.2021.711466

**Published:** 2021-09-28

**Authors:** Anna Isenhardt, Maria Kamenowski, Patrik Manzoni, Sandrine Haymoz, Cédric Jacot, Dirk Baier

**Affiliations:** ^1^Criminological Research Institute of Lower Saxony, Hanover, Germany; ^2^Institute for Penal Law and Criminology, University of Bern, Bern, Switzerland; ^3^Department of Social Work, Institute of Delinquency and Crime Prevention, Zürich University of Applied Sciences, Zürich, Switzerland; ^4^School of Social Work Fribourg, University of Applied Sciences and Arts Western Switzerland, Fribourg, Switzerland; ^5^Department of Social Work, Social Policy and Global Development, University of Fribourg, Fribourg, Switzerland

**Keywords:** identity, right-wing extremism, left-wing extremism, Islamist extremism, parenting, life events, academic performance

## Abstract

Various theoretical approaches assume that identity diffusion is an influencing factor of extremism. However, there are hardly any empirical tests on this relationship. Based on a nationwide survey of 8,317 young people in Switzerland, the study analyses whether identity diffusion is associated with right-wing extremist, left-wing extremist, and Islamist extremist attitudes. In addition, the study tests whether identity diffusion mediates the influence of family and school-related variables on extremist attitudes. The results show that identity diffusion primarily increases approval of left-wing extremist and Islamist extremist attitudes. Furthermore, identity diffusion mediates to a small extent the influence of parenting on extremist attitudes.

## Introduction

Identity diffusion, a state of identity in which persons are confused about their goals, occupations, gender roles etc. (Erikson, 1959, 1968), is discussed to be linked with various forms of deviant behavior and attitudes, so also with extremism and terrorism. For example, Schwartz et al. (2009, p. 545) state that “aimless-diffused individuals are particularly vulnerable to the allures of terrorism because terrorist ideologies are espoused with certainty, purpose, and commitment that can provide a sense of direction to a previously unguided life.” People without a fixed identity are in an aversive state; they are in search of orientation and therefore more open to extremist ideologies because they offer easy answers to complex questions.

Additionally, identity diffusion was found to be related to aggression (Dammann et al., 2011). An explanation for this relationship is that deficits in enduring feelings of ambivalence, grief, or sadness resulting from identity diffusion can increasingly lead to impulsive actions and thus also to aggression (Dammann et al., 2011). An essential characteristic of extremism is that it seeks to overcome existing political conditions through aggression and violence (Baier, 2018). Joining extremist groups can therefore enable identity diffused people to exercise violence. In this respect, too, a connection between identity diffusion and extremism can be assumed.

However, empirical studies which focus on the correlation between identity diffusion and extremism are rare. The current study provides new insights into this relationship by testing the correlation between identity diffusion and three forms of extremist attitudes (right-wing, left-wing, and Islamic) in a sample of Swiss adolescents. In addition, it is tested whether identity diffusion is a mediator of the relationships between extremist attitudes and family and school-related variables.

## Identity Development

In general, identity can be understood as the awareness of being an unmistakable individual with a life story of one's own, of showing a certain consistency in one's actions and of having found a balance between individual demands and social expectations (Abels, 2010, p. 258). Differences in individual identities become apparent in the individuals' specific characteristics or qualities as well as in their attitudes and competencies (Zimmermann, 2006).

In general, identity is a communicative construct that is produced symbolically and in exchange with other individuals (Mead, 1972). Erikson (1959, 1968) described the development of identity as a dynamic process with different phases. In each of the development phases, individuals face conflicts between the individual's attitude to oneself and to one's environment. These conflicts or crises are either the result of the rapidly changing physical and psychological experiences of individuals (Kernberg, 1978, 2006) or of the lack of integration of the external and the self-image (Clarkin et al., 2006). Therefore, the individual needs to cope with these conflicts. A successful coping leads to a consolidated identity, while an unsuccessful coping can lead to a diffusion of identity. Threats to one's identity can occur at any developmental phase and thus at any point in life, but is most massive in adolescence, where the individual searches for its place in society (Erikson, 1959, 1968). The individual has to reconcile the image that he or she has made of himself or herself with the image that others have of him or her, and wants to be recognized and accepted (Erikson, 1959, 1968).

## Identity, Identity Diffusion, and Extremism

Identity as an explaining factor of extremism is discussed in various theoretical approaches (e.g., Schwartz et al., 2009). Baier (2018, p. 9), for example, distinguishes two different paths of extremist radicalization in which identity plays a significant role. One path is characterized by the formation of identity on the basis of criminogenic socialization, which makes people susceptible to violent behavior in general and extremist violence in particular. The second path involves the questioning of the previous stable identity due to special events, whereupon a phase of searching for a new identity sets in. In line with that, Transformative Learning Theory (Wilner and Dubouloz, 2010) assumes that personal crises are the starting point of extremist radicalization. If these crises cannot be overcome with the existing resources (so-called meaning schemes), new patterns are sought that create identity. This is accompanied by an openness for extremist interpretations and offers.

Erikson (1968, p. 89) also described a link between identity and radical ideologies. Young people seem particularly vulnerable when social change appears threatening their identity, leading “to support doctrines offering a total immersion in a synthetic identity (extreme nationalism, racism, or class consciousness) and a collective condemnation of a totally stereotyped enemy of the new identity.”

The Uncertainty-Identity Theory (Hogg, 2014) assumes that people who are uncertain about themselves and their identity are motivated to identify with such groups that provide clearly defined identity, beliefs, and behavioral prescriptions. Extremist ideologies and groups make such identity offers. In addition, the Integrated Threat Theory (Stephan and Stephan, 2000), which assumes that social changes (e.g., immigration) can pose threats to identity, has been used to explain especially xenophobia and right-wing extremism. One response to a threatened identity can be the advocacy of extremism (Blanka et al., 2012). Finally, the Significance Quest Theory (Kruglanski et al., 2018) states that identity, and in particular the need to maintain a meaningful identity is an influencing factor of extremism. This “theory identifies the need for personal significance as the dominant need that underlies violent extremism” (p. 108). However, none of the theories mentioned say that identity diffusion is relevant to the process of radicalization, whereby there are various reasons to focus on this special form of identity.

Marcia (1980) distinguishes four different states of identity in adolescence. Identity diffusion in his categorization is associated with low self-esteem, while moratorium and identity achievement are associated with high self-esteem. Low self-esteem, on the other hand, can result in attempts to increase it by adopting certain attitudes or certain behaviors. In contrast to identity foreclosure, which is also associated with low self-esteem, social relationships in a state of identity diffusion are problematic. The adolescent distances himself from the parents; instead, the peers or other authorities are important. In foreclosure, on the other hand, relations with the parents are not burdened; moreover, these are more significant than relations with other people. In a comparison of all four states of identity distinguished by Marcia (1980), identity diffusion thus appears to be particularly susceptible to extremist ideologies.

A relationship between identity diffusion and extremist attitudes can also be assumed with reference to Schwartz et al. (2009) stating that identity diffusion “is characterized by the absence of personally meaningful identity commitments and by confusion about how such commitments might be formed […]. In an effort to obtain some sense of purpose, aimless-diffused individuals may attach themselves intensely to some group, expressing a willingness to unquestioningly do whatever the leaders of the group ask them to do.” Extremist ideologies provide orientation, meaning, a sense of belonging, and a feeling of uniqueness. Edelstein (2003) also refers to identity diffusion as an influencing factor of right-wing extremism. Identity diffusion can lead to what he calls “negative identity” (p. 94), which implies “replacing normal sociability with exclusion and with affiliation with criminal gangs, absorption into authoritarian group structures, or the adoption of racist ideologies.”

Furthermore, identity diffusion is related to aggression (Dammann et al., 2011), because the state of diffusion in itself is perceived as aversive and emotionally challenging, and it is associated with low self-esteem. Identity diffusion is accompanied by deficits in enduring feelings of ambivalence, sadness, or grief; it intensifies impulse actions and ultimately aggression (Dammann et al., 2011, p. 66). Part of extremist ideologies is to fight against groups classified as enemies. For persons in the status of identity diffusion, the promise to us violence against enemies could be a reason to share these positions. Another reason could be the offer of getting easy answers to complex questions, which are part of many extremist ideologies. Thus, the shift toward extremist ideologies promise a rather easy way of a new identity. In line with these assumptions, the current study empirically analyses the relationship between identity diffusion and extremist attitudes. In the study, not only one form of extremist attitudes are considered. Instead, the relationship between identity diffusion and extremism is analyzed for right-wing, left-wing, and Islamist extremist attitudes in a sample of adolescents. Therefore, the current study refers to the period of life where the development of one's own identity takes place and young people are most open to the identification with negative identity offerings. Based on the different theoretical considerations Hypothesis 1 is as follows:

H1: Increasing levels of identity diffusion lead to an increase of right-wing, left-wing, and Islamist extremist attitudes.

## Right-Wing, Left-Wing, and Islamist Extremism

Usually, extremism is characterized by the fact that it rejects the democratic constitutional state and wants to eliminate or restrict its constitutional (separation of powers, protection of fundamental rights) and democratic components (popular sovereignty, human fundamental equality; Goertz and Goertz-Neumann, 2018, p. 11). On a very general level, therefore, all forms of extremism have one thing in common: they seek to abolish the democratic order and introduce a different socio-political order in its place. A second common feature is that all extremist ideologies, with regard to the different socio-political order they seek to establish, make a strong distinction between their own group and one or more out-groups; at the same time, these out-groups are declared enemies of the new socio-political order they seek to establish. The various forms of extremism then differ considerably with regard to the concrete form of the new order and which groups are defined as enemies. The third common feature of all forms of extremism is that in order to achieve their ideological goals they accept the use of violence (Baier, 2018).

One common form of political extremism is right-wing extremism, aiming at introducing a nationalist socio-political order and distinguishing between the national own group and numerous foreign groups. This strong distinction between different population groups is called Social Darwinism. Other “races,” foreigners in general, Muslims, and Jews are classified as out-groups, which is why racism, xenophobia, Islamophobia, and anti-Semitism are important parts of the right-wing extremist ideology. Consequently, violent acts are directed against these groups as well as against political opponents, namely from the left side of the political spectrum (Manzoni et al., 2018; Bjørgo, 2019).

A second form of political extremism is *left-wing extremism*. As a socio-political order it aims at the introduction of communism or anarchism. The enemy images of these orientations are on the one hand capitalism and on the other hand the state and its organs classified as repressive, especially the police. Although the police are also an enemy in other extremist ideologies, it is true for left-wing extremism that the police are at the very top of the ranking of enemy images (Schroeder and Deutz-Schroeder, 2015, p. 453). Out-group members of this form of extremism are capitalists, police officers and right-wing extremists (Manzoni et al., 2018). In left-wing extremism, it is therefore permitted or even necessary to use violence against these out-group members.

The central ideological goal of the last form of extremism analyzed here, *Islamist extremism*, is the introduction of a theocracy based on the Quran and the Sharia as a new socio-political order (Goertz and Goertz-Neumann, 2018); the introduction of a theocracy clearly distinguishes this form from other forms of extremism such as right-wing extremism, although there may well be overlaps in other areas (e.g., anti-Semitism)—this justifies considering Islamism as its own form of extremism. Islamist extremism is a form of political and not only religious extremism, which becomes obvious by regarding its political goal, which contain, inter alia, the liberation of Islamic states, expulsion of occupying powers from the west, and particularly the establishment of an Islamic state. The own group of right-believing Muslims is upgraded; the West in general and non-Muslims as well as non-traditional Muslims in particular are regarded as out-groups. In addition, the inhabitants of the western country in which the Muslims live are classified as an out-group. The willingness to use violence is, on one hand, directed against non-Muslims. On the other hand, terrorist attacks are seen as central means of achieving the goal of Islamist extremism (Vidino, 2013; Manzoni et al., 2018).

## Influencing Factors of Identity Diffusion and Extremism

The number of possible influencing factors of identity diffusion is large, and of extremism, it is even larger. Therefore, it is not possible at this point to provide a comprehensive discussion of all these influencing factors. Instead, we will concentrate mainly on the area of the family and the school.

Referring to Erikson's developmental theory, Edelstein (2003, p. 92) argues that family experiences are central to the formation of identity diffusion: “a destructive family dynamic, for example an authoritarian and repressive relationship […] may threaten the process of identity formation and put […ones] at risk of identity diffusion or disintegration.” In line with that, Igarashi et al. (2009) found for Japanese university students that parental neglect as well as punishment were positively correlated with identity diffusion. If a form of parental education oriented toward obedience, oppression and punishment can reinforce identity diffusion, then it can be assumed, with regard to the educational style concept of Baumrind (1989), that *authoritative parenting* in particular should prevent identity diffusion. This form of parenting is characterized by the fact that parents both monitor their child's behavior and give emotional care. Various studies have shown that the two educational style dimensions of monitoring and care protect children and adolescents from developing in a problematic manner (Leschied et al., 2008, Hoeve et al., 2009). Also, in relation to extremism it is reported that “at the family level, an appreciative/positive parenting behavior […] had a protective effect on different kinds of extremism” (Lösel et al., 2018, p. 95). If both identity diffusion and extremism are reduced by positive parenting behavior, it can be assumed that the effect of parenting is at least partially mediated by identity diffusion: Because a positive upbringing leads to less identity diffusion, it also has a protective effect on extremism.

In addition to authoritative parenting, the empirical analyses will also consider *parental inconsistency*. This implies that children are brought up in contradictory ways. As a result, the predictability of parental behavior is low. Jaursch et al. (2009) and Hoeve et al. (2009), among others, were able to show that this parenting style is associated with problem behavior and delinquency of children and adolescents. Although there are no findings to date on the relationship between parental inconsistency and extremism, it can be assumed that this educational style also increases extremist attitudes. It can also be hypothesized that parental inconsistency influences identity diffusion, insofar as children and adolescents are confronted with contradictory information, expectations, norms, etc. on the part of their parents.

An additional influential factor of both identity diffusion and extremism is the experience of *critical life events*. Events as the divorce of parents, the death of one parent, a serious illness of one's own, etc. are crisis-like events that call the previous identity into question. The crisis situation triggers an identity crisis, the search for a new orientation, for meaning. An identity-related opening for new ideas is taking place (Baier, 2018). Extremism research has repeatedly examined the influence of such crisis experiences. According to the aforementioned Transformative Learning Theory (Wilner and Dubouloz, 2010) they can form the basis of radicalization. Sikkens et al. (2017) conclude in their study, that there is an impact of negative family experiences: The majority of the families they studied were affected by divorce, father's absence, illness, or deaths. These events may have reduced the level of attention and control over children's development so that parents were unable to respond adequately to problematic biographical changes of the children.

Extra-family factors also have an influence on identity diffusion and extremism. With the exception of one factor, however, they are not considered in the following analyses. One school-related factor is included, namely *academic performance*. The school context is seen as very important for identity development (Lannegrand-Willems and Bosma, 2006). With regard to extremism, Lösel et al. (2018) state on the basis of their review, “good school achievement […] reduced far-right and far-left extremist attitudes and behavior” (p. 95). A low level of success at school can also be seen as a form of critical life event that can lead to identity diffusion. Meeus (1993), among others, was able to find support for the relationship between school success and identity development.

If the mentioned findings on family and school performance are summarized, Hypothesis 2 can be formulated as follows:

H2: Authoritative parenting reduces identity diffusion and extremism. Parental inconsistency, critical life events and poor academic performance, on the other hand, increase identity diffusion and extremist attitudes. Identity diffusion partially mediates the influence of these influencing factors on extremist attitudes.

In addition, the analyses take into account various control variables that can be assumed to be related to both identity diffusion and extremism. These include gender (e.g., Cramer, 2000; Davies, 2008), socio-economic status (e.g., Phillips and Pittman, 2003; Schmid, 2013) and ethnic origin (Thomas et al., 2016; Koser and Cunningham, 2018).

## Method

### Procedure

A sample of young people living in Switzerland is used to test both hypotheses. The aim of the underlying study was to determine the prevalence and influencing factors of three forms of political extremism in Switzerland. In order to get the sample, the following procedure was used: The survey did not claim to be representative for whole Switzerland, as this would have been very difficult to achieve for a total of 26 cantons. Instead, the survey was conducted in ten cantons, with urban and more rural as well as German-, French- and Italian-speaking cantons being included. The survey focused on young people aged between 17 and 18 on average. Accordingly, all types of schools in the ten cantons in which young people of this age are taught were included in the random sampling (vocational school, transitional education, grammar school, and technical/commercial secondary school). In the cantons, a random drawing of schools or school classes was then carried out in which surveys were to be conducted.

Students were provided with an online questionnaire during one school lesson (45 min); the surveys were administered by trained interviewers or teachers. During the survey, a class work atmosphere was created, i.e., the pupils were, for example, set apart and it was ensured that they completed the questionnaire in a disciplined manner. Anonymity and confidentiality was guaranteed. The parents of the students were informed with a letter before the survey and could give their veto to their children's participation. The students could also decide for themselves whether or not they wanted to take part in the survey. Data collection lasted from 4/24/2017 to 12/21/2017.

### Sample

Five hundred ninety-five classes with all in all 8,317 students took part in the survey; 232 schools were originally approached, of which 123 ultimately participated in the survey. The total response rate of the survey was 39.1%. This is a relatively low rate because many schools refused to take part in the survey. If schools or classes agreed to participate, nine out of ten pupils took part in the survey.

The age of the respondents was asked in categories from “under 16 years” to “21 years and older,” therefore the exact age distribution of the sample cannot be reported. However, it can be stated that 83.7% of the respondents are between 16 and 19 years old. Only 3.1% of respondents reported being under 16 years old; 4.7% of respondents are 20 years, 8.6% of respondents are 21 years and older. Overall, therefore, a largely age-homogeneous group of young people between 16 and 19 was reached.^1^ The gender ratio of the survey is balanced (male youths 49.7%, female youths 50.3%). Of all respondents, 52.0% attend vocational school, 12.3% attend technical/commercial secondary school, 26.4% attend a grammar school and 9.3 % undergo transitional training. Most students were born in Switzerland, 16.9% of the students were not born in Switzerland (the five most frequent mentioned countries were: Portugal, Italy, Germany, France, Kosovo).^2^ In total, 44.7% of respondents come from rural areas (under 5,000 inhabitants), 37.6% from small towns (under 20,000 inhabitants), 17.7% from cities (over 20,000 inhabitants).

### Measures

*Identity diffusion* was measured using items of the Inventory of Personality Organization (IPO) which is based on Kernberg (1978, 2006). The inventory includes 21 items that capture identity diffusion. According to the study by Igarashi et al. (2009), five items with high factor loading or high validity were selected. The items are “1. My life goals change frequently,” “2. I pick up interests and then drop them,” “3. I see myself in different ways at different times,” “4. My tastes and opinions are borrowed from other people,” and “5. People cannot guess how I'm going to behave.” The answering scale reached from “1–not true at all” to “6–completely true.” The five-item scale has a Cronbach's α of 0.73 and the selectivity of the items is at least 0.35. Igarashi et al. (2009) used the scale on an older, primarily student population, not on an adolescent population. This is a disadvantage, although the validity of the selected five items may be given. However, there are no established instruments for assessing identity diffusion, which is why this instrument was used.

In accordance with the definitions of extremism presented above, the three forms of extremist attitudes were measured using multi-item instruments which are based on existing instruments or have been partly newly developed (see Kamenowski et al., 2021 for references). All items are listed in the Appendix. *Right-wing extremism* was measured with ten items, with six of them measuring approval of ideological positions, with one item recording approval of the new socio-political order of nationalism and five items measuring the devaluation of out-groups. In addition, four items were taken into account that contain a positive attitude toward the use of violence against out-groups, whereby on the one hand the out-group of foreigners in general and on the other hand the out-group of left-wing extremists were considered (and verbal as well as physical violence). In total, the scale for measuring right-wing extremist attitudes comprises ten items, which could be agreed or disagreed from “1–not true at all” to “6–completely true.” The reliability of the scale amounts to Cronbach's α. = 0.88; the selectivity of the items is at least 0.44.

*Left-wing extremism* was measured with nine items. Two items measure the approval of the establishment of a communist/anarchist socio-political order, three items measure the devaluation of out-groups (capitalists, state/police). A further four items measure approval of the use of violence, which is directed at political opponents (right-wing extremists) on the one hand and capitalists or state actors (police) on the other. All items could be agreed or disagreed from “1–not true at all” to “6–completely true.” The reliability of the scale amounts to Cronbach's α = 0.79; the selectivity of the items is at least 0.33.

Eleven items were used to measure *Islamist extremism*. Seven items measure the degree of agreement with ideological goals of this extremism (two items: new socio-political order of theocracy, five items: revaluation of the self and devaluation of out-groups). Four items in turn measure the approval of using violence, which can be directed against non-Muslims (two items) or includes terrorism or the armed struggle of the Islamic state (two items). All items could be agreed or disagreed from “1–not true at all” to “6–completely true.” The reliability of the scale amounts to Cronbach's α = 0.81; the selectivity of the items is at least 0.36.

As the means listed in the tables in the Appendix show, all statements are agreed to a rather low level. The items on ideological positions are more strongly agreed than the items on the use of violence. Moreover, it is shown that items that measure left-wing extremism are more strongly supported than items on right-wing extremism and Islamist extremism.^3^

Two different parenting styles are analyzed in the current study. First, *parental inconsistency* is measured by three items adopted from Krohne and Pulsack (1995). The items for an inconsistent parenting were “My (step-)parents announced something (e.g., a trip) and then dropped it in the water,” “My (step-)parents scolded me when I did not expect it at all,” and “My (step-)parents promised me to bring something, but then didn't do it.” Second*, parental monitoring* as a component of authoritative parenting was measured with the following three items: “My (step-)parents knew where I was when I wasn't at home,” “My (step-)parents knew what I was doing when I wasn't at home,” and “My (step-)parents knew which friends I was with when I was not at home.” All items measuring parenting were related to the past in the formulation of the question (“Please think about parenting by your (step-)parents. How often did this happen in the past?”) and students were asked how often the different forms of parental behavior happened. To answer the items, a 5-point scale reaching from “1–never” to “5–very often” were provided. Scale reliabilities are Cronbach's α = 0.63 for inconsistency (selectivity at least 0.38) and α = 0.85 for monitoring (selectivity at least 0.70).

To measure *critical life events* the experience of five different forms of personal crises have been queried, namely divorce or separation of parents, serious disease of a close person, serious disease of oneself, death of father or mother, and moving with loss of previous social contacts. All five life events were coded dichotomously (0–not experienced, 1–experienced). The five items were summed up to an index that can have values between 0 (no critical life event experienced) and 5 (all events experienced). On average, students reported 0.97 critical life events.

*Academic performance* was measured with an item whose wording was “How well do you assess your school performance?” The answer categories ranged from “1–excellent, I probably belong to the best” to “7–poor, I probably belong to the worst.” The mean value of the sample is 3.34.

The variables sex (0–male, 1–female), country of birth (0–born in Switzerland, 1–born abroad^4^) and socio-economic status are used as control variables in the analyses. To measure status, the young people should rate the following statement (from “1–very poor” to “5–very good”): “How do you manage with the money (pocket money, money gifts, money you earn yourself) that you personally have at your disposal each month?” High scores thus stand for a high status. Other, more common status variables (e.g., income or occupational status of parents) were not measured in the survey.

### Analytical Strategy

The relationships between the presented variables are subsequently examined by means of structural equation models, whereby the program Mplus 7.31 (Muthén and Muthén, 2015) was used. Using structural equation modeling allows to specify measurement models (latent variables) and structural models (relationships between latent variables). In a first step, the different measurement models were analyzed. After that, the different structural models were estimated. In accordance with the conventions, latent variables are shown as ellipses in the following figures, manifest variables as rectangles. In accordance with the previous explanations, the latent variables were recorded with three to eleven items.

## Results

**Figure 1:**
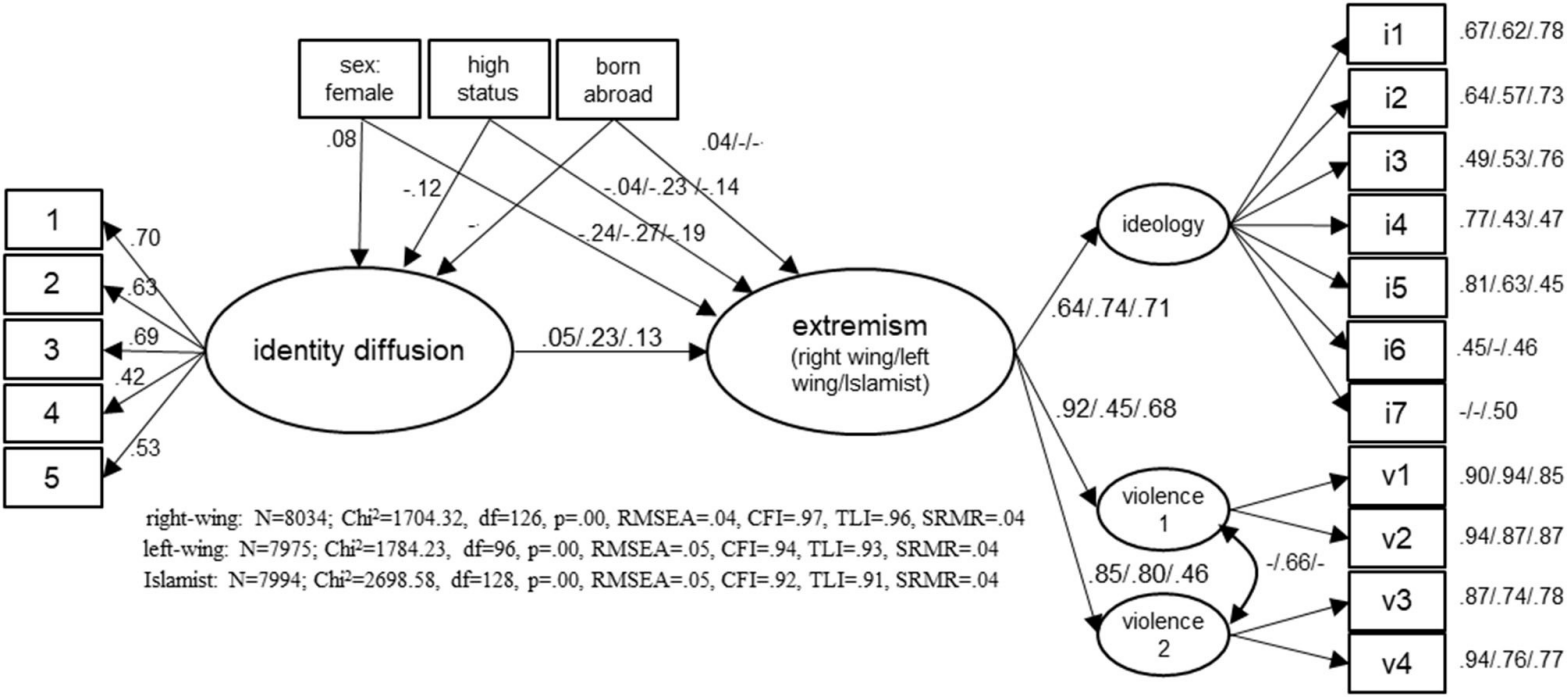
Model to explain extremist attitudes by identity diffusion. Displayed: standardized coefficients; estimation procedure: Maximum Likelihood; order of displayed coefficients: left coefficient: right-wing extremism/medium coefficients: left-wing extremism/right coefficients: Islamist extremism; all coefficients significant at *p* < 0.05; “–” coefficient not significant at *p* < 0.05.

The relationship between identity diffusion and extremist attitudes (Hypothesis 1) was tested using three separate structural equation models, one for each form of extremist attitude. The results of the different models including the model-fit values are summarized in Figure 1 (standardized coefficients are shown). All models show a sufficient fit (Hu and Bentler, 1999); this means that the theoretical assumptions specified in the models fit well with the empirical observations. The fact that the Chi^2^ statistics is significant (indicating a less good fit between theory and data) is due to the large sample. For this reason, this parameter should not be considered a relevant criterion for acceptance or rejection of the model, but other parameters such as the RMSEA and SRMR (good fit: ≤ 0.05) or the CFI and TLI (sufficient fit: ≥0.90) should be used for evaluation.

If the measurement model for identity diffusion is considered, we can speak of a reliable model, whereby the factor loading of the fourth item (λ = 0.42) is somewhat lower than the loadings of the first three items in particular. To model extremist attitudes, a second-order factor model was specified. The first-order factors measure ideological goals and approval of violence; in the case of approval of violence a distinction is made between the different out-groups against which violence may be carried out (e.g., right-wing extremism: foreigners in general and left-wing extremists). The three first-order factors are then combined to form the second-order factor “extremist attitude.” In the model on left-wing extremist attitudes, this results in a strong error correlation between the two factors measuring approval of violence, which was taken into account in the model specification. In the other two models there is no significant error correlation between the two violence-approval-factors, which is why this was not modeled. All non-significant correlations in the model were deleted when the models were calculated and marked with “–” in Figure 1. Since the ideology dimension consists of six to eight items, depending on the form of extremism, there is no loading for some items shown in Figure 1. For reasons of clarity, the loadings of the individual items were not listed directly on the paths, but next to the items. For all items there are sufficiently high loadings on first-order factors. For each form of extremist attitudes, these in turn load sufficiently on the second-order factors, although the loadings do vary (0.45 ≤ λ ≤ 0.92).

The structural model indicates a significant correlation between identity diffusion and all forms of extremist attitudes. Identity diffusion significantly increases agreement to extremist attitudes. However, the different forms of extremist attitudes differ considerably: A strong correlation is found for left-wing extremist attitudes (γ = 0.23). A negligible correlation, which is however shown to be significant due to the sample size, is found for right-wing extremist attitudes (γ = 0.05). The effect on Islamist extremist attitudes lies in between (γ = 0.13).

All in all, the influence of the control variables on identity diffusion and extremist attitudes is low. Female respondents and respondents with a lower status show a higher level of identity diffusion; respondents born abroad do not differ in terms of identity diffusion from respondents born in Switzerland. Moreover, the variable born abroad is largely uncorrelated with extremist attitudes. In contrast, female respondents significantly less frequently agree with all forms of extremist attitudes. It is also true that respondents with a higher status are less likely to agree with all forms of extremism. This effect is lowest for right-wing extremist attitudes.

**Figure 2:**
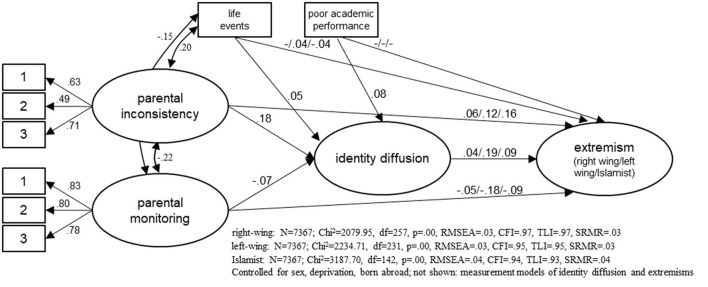
Model to explain extremist attitudes by identity diffusion and other independent variables. Displayed: standardized coefficients; estimation procedure: Maximum Likelihood; left coefficients: model for right-wing extremism/medium coefficients: left-wing extremism/right coefficients: Islamist extremism; all coefficients significant at *p* < 0.05; “–” coefficient not significant at *p* < 0.05.

The correlations expected in Hypothesis 2 were tested using extended structural equation models. The results are presented in extracts in Figure 2. We have refrained from reproducing the measurement models for identity diffusion and extremist attitudes that are already known from Figure 1. Also not shown are the control variables and their correlations with the newly included influencing factors. The models for all three forms of extremism in turn have sufficient fit values (see Figure 2); RMSEA, SRMR, CFI, and TLI values all have acceptable levels.

All four independent variables are related to identity diffusion, as assumed in Hypothesis 2. With the exception of parental inconsistency, however, the correlations are weak. Parental monitoring reduces identity diffusion (γ = −0.07); parental inconsistency (γ = 0.18), the experience of critical life events (γ = 0.05) and poor school performance (γ = 0.08) are associated with higher identity diffusion.

According to the results, extremist attitudes do not depend on poor school performance—no significant correlations are found for any form of extremism. The experience of critical life events is also only negligibly related to extremist attitudes (slightly increases left-wing extremist attitudes, slightly reduces Islamist extremist attitudes). With regard to the two educational styles, there are different correlations—depending on the form of extremism. Right-wing extremist attitudes are only slightly increased by parental inconsistency (γ = 0.06) and reduced by parental monitoring (γ = −0.05). For the other two forms of extremist attitudes, however, there are stronger risk-increasing effects of parental inconsistency (left-wing extremist attitudes: γ = 0.12, Islamist extremist attitudes: γ = 0.16) or protective effects for parental monitoring (left-wing extremist attitudes: γ = −0.18, Islamist extremist attitudes: γ = −0.09). Even when all factors in the model are taken into account, the correlations between identity diffusion and extremist attitudes are only slightly reduced.

In addition, some correlations had to be allowed between the independent variables in the model to obtain an acceptable model fit. Correspondingly, it is shown in Figure 2 that the experience of critical life events is associated with higher parental inconsistency (*r* = 0.20) and lower parental monitoring (*r* = −0.15). In addition, parental inconsistency and parental monitoring correlate negatively (*r* = −0.22).

To assess whether identity diffusion mediates the effects of academic performance, life events, and parental styles on extremist attitudes indirect effects were calculated (see Table 1). They are composed of the product of the effect from the aforementioned variables on identity diffusion and of the effect from identity diffusion on either form of extremist attitudes. For example, the effect from poor academic performance on identity diffusion is γ = 0.08 and the one from identity diffusion on right-wing extremist attitudes is γ = 0.04. Thus, the indirect effect is γ = 0.0032. Rounded, this gives an effect of γ = 0.00. However, despite its small size, this effect, as some other small effects, is significant on a level of 0.05, which is due to the large sample size, whereby even very small, actually rarely relevant effects can become significant.

**Table 1:** Indirect effects via identity diffusion.

	**Right-wing extremist attitudes**		**Left-wing extremist attitudes**		**Islamist extremist attitudes**	
	**Indirect**	***p***	**Indirect**	***p***	**Indirect**	***p***
Poor academic performance	0.00	0.026	0.02	0.000	0.01	0.000
Life events	–	–	0.01	0.002	0.00	0.008
Parental inconsistency	0.01	0.019	0.03	0.000	0.02	0.000
Parental control	−0.00	0.036	−0.01	0.000	−0.01	0.001

*Displayed: standardized coefficients; estimation procedure: Maximum Likelihood*.

The indirect effects are generally low. This is especially true for right-wing extremist attitudes. For left-wing extremist attitudes, small significant mediating effects of identity diffusion can be observed for all four variables, for Islamic extremist attitudes for poor academic performance, parental inconsistency, and parental control. The indirect effects of academic performance on left-wing and Islamic extremist attitudes are significant, while the direct effects are not. Therefore, left-wing and Islamic extremist attitudes are only affected by school performance when mediated by identity diffusion. For the parenting variables, in particular parental inconsistency, there are mediating effects, but the direct effects on extremist attitudes are ultimately stronger.

## Discussion

According to different theoretical approaches, identity diffusion can be assumed as a cause of extremist radicalization. In particular, it can be assumed that young people who are insecure about their identity are at risk to adopt extremist ideologies to create a stable identity because they offer a clear world view, orientation, and security. The current study empirically tests this relationship for three different forms of extremist attitudes (right-wing, left-wing, and Islamic) in a sample of adolescents in Switzerland. Results indicate an empirical correlation between identity diffusion and extremist attitudes (Hypothesis 1), which is strongest for left-wing extremist attitudes. The correlation with right-wing extremist attitudes is rather low. This difference seems to be hard to explain. Right-wing extremism presents a closed world view including a strong differentiation between native in-group and different out-groups (e.g., foreigners, jews, etc.), what should be attractive to people with a diffused identity. However, results show that left-wing, and to a smaller extent also Islamic extremist attitudes, are more attractive to people in a diffused identity status. First, it must be noted that some of the measurement instruments represent new developments; thus, the results may be attributed to measurement instruments that are still insufficiently established, especially with regard to left-wing extremist and Islamist extremist attitudes. Secondly, theoretical aspects may also be of importance, although we can only speculate in this regard due to the scarcity of research to date. A potential reason why left-wing extremism is more strongly correlated with identity diffusion could be that it is diffused itself. The aims of this form of extremism and its out-group concept are less pronounced. Left-wing extremism has different ideological foundations, subcultures and fields of action (cf. Pfahl-Traughber, 2020, among others), which could make it attractive to individuals with different (or diffused) needs.

Next to the analyses of the correlation between identity diffusion and extremist attitudes the study investigates the influence of different factors on identity diffusion. In relation to Hypothesis 2 results show that the effects of parental styles, critical life events, and academic performance are in the expected direction. However, correlations are rather weak except for parental inconsistency, which increases identity diffusion. With regard to school grades, it can be assumed that these represent only one area of school factors that could be less important for attitudes and behaviors in higher grades—and the young people studied here were in higher grades or had already completed school (and were attending vocational school)—than in younger grades. This does not mean that other school factors not studied here, such as school attachment, social integration into the class, etc., might be significant; further analyses of how school factors relate to identity and extremism are therefore desirable.

Additionally, critical life events do not affect young peoples' identities. This means that experiencing critical life events does not *per se* trigger identity diffusion. However, it is probably important how these events can be handled and which kind of personal and social resources are available to deal with these events. Finally, the effect of authoritative parenting cannot be fully assessed based on the results of the current study, because just one dimension, parental monitoring, was analyzed here. Future studies should include also other aspects of this parenting style, for instance, parental care. Results presented her show, that parental control alone does not prevent developing identity diffusion.

Additionally, based on Hypothesis 2 it was tested whether and to what extend identity diffusion functions as a mediator in the relationship between the familial and school variables and extremist attitudes. It can be stated first, that poor academic performance and critical life events have only small or no correlation with extremist attitudes and only small mediating effects can be observed. However, when these variables affect extremist attitudes, they affect them via identity diffusion. In addition, critical life events are not *per se* risk factors for radicalization. They increase identity diffusion to a small extent and also slightly extremist attitudes. The fact that critical life events contribute so little to the explanation of identity diffusion and extremism could be related to the fact that they are processed very differently from person to person, for example also in an internalizing form.

For the parenting variables, empirical results meet the assumptions formulated in Hypothesis 2. Parental inconsistency increases extremist attitudes, parental control reduces them. These effects are partly mediated by identity diffusion. Thus, it can be concluded, that identity diffusion is a mechanism that helps to understand and explain in which way family factors influence radicalization. Next to other factors, they form the identity status of young people and when this status is diffused, extremist ideologies are more easily accepted. Again, it has to be stated, that these correlations are weaker for right-wing extremism in comparison to the other two forms. Therefore, this form of extremism seems in some ways to be special. This may partly be explained by the status of right-wing ideologies in Switzerland. For instance, national thinking and a certain national pride have a long tradition in Switzerland and are not perceived as negative *per se*. Parts of the extreme right-wing ideology are possibly more accepted here than in other countries. Acceptance of this ideology is then less an expression of maladaptive development processes (such as identity diffusion), but at least up to a certain point part of the normal process of identity formation. Accordingly, Marcia (1980) suggests that the status of “identity achievement” correlates with right-wing extremism, not the status of identity diffusion. However, future studies would need to test this assumption.

The study has some limitations that should be mentioned at the end. For example, it is only a cross-sectional study, which does not permit any conclusive statements about cause-and-effect relationships. The response rate of the survey is below average for school-class based surveys, so that it cannot be ruled out that the groups ultimately reached are in some way selective. In addition, all data are based on self-reports; especially the ratings on extremist attitudes cannot be validated by other sources. With regard to the measuring instruments used, it should be mentioned as a limitation that, firstly, extremist attitudes were surveyed with partly newly developed instruments, which have to be validated in further studies. Secondly, identity diffusion was measured by a short scale consisting of only five items which were originally used on an older group of people. The results of the study should therefore be checked with more extensive and validated measuring instruments.

## Data Availability Statement

The data for all analyses can be accessed here: https://osf.io/wy8g7.

## Ethics Statement

The studies involving human participants were reviewed and approved by Cantonal Ethics Commission of the canton of Zurich. Written informed consent from the participants' legal guardian/next of kin was not required to participate in this study in accordance with the national legislation and the institutional requirements.

## Author Contributions

All authors listed have made a substantial, direct and intellectual contribution to the work, and approved it for publication.

## Funding

The research was funded by the Swiss National Science Foundation under grant number 165760.

## Conflict of Interest

The authors declare that the research was conducted in the absence of any commercial or financial relationships that could be construed as a potential conflict of interest.

## Publisher's Note

All claims expressed in this article are solely those of the authors and do not necessarily represent those of their affiliated organizations, or those of the publisher, the editors and the reviewers. Any product that may be evaluated in this article, or claim that may be made by its manufacturer, is not guaranteed or endorsed by the publisher.

## Footnotes

^1^Extremist attitudes may not be most prevalent in this age group analyzed here. Existing analyses of right-wing extremism, however, show that right-wing extremist attitudes can also be found among younger people (aged 18 and older), although older people are even more likely to agree with right-wing extremist attitudes (Rippl, 2005). Existing studies on left-wing extremist attitudes, however, confirm that approval is higher among young people than among older people (Treskow and Baier, 2020, p. 29).

^2^The proportion of young people with a migration background is 52.1%, which is not surprising given the fact that Switzerland is a country of immigration. In order to operationalize the migration background, the adolescents were asked to indicate in which country the biological mother and father were born. If at least one parent was not born in Switzerland, then a migration background was assumed. The largest group of young people with a migration background is formed by Portuguese young people (6.6% of the total sample). The second largest migrant group consists of young people of whom at least one parent was born in Italy (5.7%), and the third largest group consists of young people of whom at least one parent was born in Kosovo (4.4%).

^3^It may be questionable whether all statements can be answered by all respondents, insofar as, for example, the items measuring Islamist extremism primarily refer to Muslims. However, individual statements may very well be agreed with by young people from other religious backgrounds (e.g., devaluation of western societies); moreover, young people for whom these statements do not apply were able to answer “do not agree.” It therefore seems appropriate that all scales are analyzed for all adolescents.

^4^The variable “born abroad” was used for the analysis, not, for example, “migration background,” because this implies that people have had migration experiences themselves, which can be assumed to represent some kind of critical life event. A migration background, on the other hand, means that people did not migrate themselves, but that it was usually their parents who had migration experiences.
