# Supplementary material for: Identity Diffusion and Extremist Attitudes in Adolescence
Source: Front Psychol. 2021 Sep 28;12:711466. doi: 10.3389/fpsyg.2021.711466 (PMC8505980; doi:10.3389/fpsyg.2021.711466)
Supplement: Supplementary file 1 [file Data_Sheet_1.docx]

# Appendix

**Table A1: items of the right-wing extremism attitude scale**

|  | **mean** | **std.dev** |
| --- | --- | --- |
| i1. Switzerland should belong only to the Swiss, who have already lived here for many generations. (nationalism) | 2.24 | 1.45 |
| i2. The Swiss are innately superior to other nations. (social Darwinism) | 2.07 | 1.36 |
| i3. The Whites are world leaders, and rightly so. (rassism) | 2.03 | 1.37 |
| i4. If there are not enough jobs, the foreigners living in Switzerland should be sent back to their home country. (xenophobia) | 2.25 | 1.45 |
| i5. Immigration into Switzerland should be forbidden for Muslims. (islamophobia) | 2.08 | 1.40 |
| i6. Jews have too much power in Switzerland. (antisemitism) | 1.85 | 1.26 |
| v1. I think it is okay to beat up foreigners in Switzerland because they are foreigners. (willingness to use violence against foreigners) | 1.39 | 0.93 |
| v2. I think it is okay to verbally harass foreigners because they are foreigners. (willingness to use violence against foreigners) | 1.45 | 1.00 |
| v3. I think it is okay if the meeting points or houses of left-wing extremists, members of the Black Block, squatters, punks and the like are demolished. (willingness to use violence against left-wing extremists) | 1.61 | 1.12 |
| v4. I think it is okay if left-wing extremists, members of the Black Block, squatters, punks or the like are beaten up due to their political views. (willingness to use violence against left-wing extremists) | 1.55 | 1.06 |

**Table A2: items of the left-wing extremism attitude scale**

|  | **mean** | **std.dev** |
| --- | --- | --- |
| i1. We will really only be free, if the whole state is abolished. (communism/anarchy) | 1.97 | 1.29 |
| i2. We do not need a state and no parties; we are the best at governing ourselves. (communism/anarchy) | 1.95 | 1.17 |
| i3. The business leaders destroy the habitat of the simple people in the cities. (hostility towards capitalism) | 2.86 | 1.37 |
| i4. The major companies around the world are responsible for poverty and hunger in the world. (hostility towards capitalism) | 3.39 | 1.43 |
| i5. The police and state only protect the rights of the rich. (hostility towards the police and the state) | 2.51 | 1.34 |
| v1. I think it is okay to beat up right-wing extremists, xenophobes, fascists, Nazis or the like because of their political views. (willingness to use violence against right-wing extremists) | 1.86 | 1.31 |
| v2. I think it is okay to demolish the meeting points of right-wing extremists, xenophobes, fascists, Nazis or the like. (willingness to use violence against right-wing extremists) | 2.01 | 1.43 |
| v3. I think it is okay if the buildings or luxury cars of the major companies and business leaders around the world are damaged. (willingness to use violence against capitalists) | 1.68 | 1.13 |
| v4. I think it is okay to use violence against the police (e.g. blows, kicks, rocks, pyros). (willingness to use violence against police officers) | 1.61 | 1.11 |

**Table A3: Items of the Islamist extremism attitude scale**

|  | **mean** | **std.dev** |
| --- | --- | --- |
| i1. The Swiss society must be reformed according to Islamic rules. (introduction of theocracy and Sharia) | 1.33 | 0.82 |
| i2. The Islamic laws of Sharia, whereby adultery or homosexuality are severely punished, for example, are much better than the Swiss laws. (introduction of theocracy and Sharia) | 1.47 | 1.01 |
| i3. Islam is the one true religion; all other religions are of lesser value. (superiority of Islam) | 1.30 | 0.87 |
| i4. I am repulsed by the lifestyle of people in the western world (e.g. wearing expensive clothing, open sexuality). (devaluation of western societies) | 2.03 | 1.26 |
| i5. Those who do not literally follow the rules of the Koran are not real Muslims. (hostility towards non-traditional Muslims) | 1.89 | 1.29 |
| i6. Those who change the original Islam are betraying Islam. (hostility towards non-traditional Muslims) | 2.07 | 1.42 |
| i7. The Swiss Christians are of less value than the Muslims here. (hostility towards Swiss) | 1.53 | 0.99 |
| v1. I think it is okay to verbally harass people who do not belong to Islam as sceptics or the like. (willingness to use violence against non-Muslims) | 1.35 | 0.86 |
| v2. I think it is okay if Muslims are physically punished because they did not abide by the religious rules. (willingness to use violence against non-Muslims) | 1.38 | 0.93 |
| v3. I think it is okay if Muslims fight for their cause with violence and terrorist attacks. (Advocacy of terrorism/IS) | 1.42 | 1.06 |
| v4. I think it is okay if young people go abroad to fight for the Islamic state or other Islamic groups. (Advocacy of terrorism/IS) | 1.50 | 1.08 |
